# Folium Sennae Increased the Bioavailability of Methotrexate through Modulation on MRP 2 and BCRP

**DOI:** 10.3390/ph14101036

**Published:** 2021-10-12

**Authors:** Chung-Ping Yu, Yu-Hsuan Peng, Ching-Ya Huang, Yow-Wen Hsieh, Yu-Chi Hou, Shiuan-Pey Lin

**Affiliations:** 1 School of Pharmacy, China Medical University, No. 100, Sec. 1, Jingmao Rd., Beitun Dist., Taichung 406040, Taiwan; yu1095813@gmail.com (C.-P.Y.); pengayu@gmail.com (Y.-H.P.); chingya301@gmail.com (C.-Y.H.); yowenhsieh@gmail.com (Y.-W.H.); 2 Department of Pharmacy, China Medical University Hospital, No. 2, Yude Rd., North Dist., Taichung 404332, Taiwan; 3 Department of Pharmacy, Asia University Hospital, Taichung, No. 222, Fuxin Rd., Wufeng Dist., Taichung 413505, Taiwan; 4 College of Medical and Health Science, Asia University, No. 500, Lioufeng Rd., Wufeng Dist., Taichung 41354, Taiwan

**Keywords:** constipation, Folium Sennae, methotrexate, multidrug resistance-associated proteins, breast cancer resistance protein

## Abstract

Folium Sennae (FS), a popular laxative (Senna), contains polyphenolic anthranoids, whose conjugation metabolites are probable modulators of multidrug resistance-associated proteins (MRPs) and breast cancer resistance protein (BCRP). We suspected that the combined use of FS might alter the pharmacokinetics of various medicines transported by MRPs or BCRP. This study investigated the effect of FS on the pharmacokinetics of methotrexate (MTX), an anticancer drug and a probe substrate of MRPs/BCRP. Rats were orally administered MTX alone and with two dosage regimens of FS in a parallel design. The results show that 5.0 g/kg of FS significantly increased the AUC_0–2880_, AUC_720–2880_ and MRT of MTX by 45%, 102% and 42%, and the seventh dose of 2.5 g/kg of FS significantly enhanced the AUC_720–2880_ and MRT by 78% and 42%, respectively. Mechanism studies indicated that the metabolites of FS (FSM) inhibited MRP 2 and BCRP. In conclusion, the combined use of FS increased the systemic exposure and MRT of MTX through inhibition on MRP 2 and BCRP.

## 1. Introduction

Constipation is a common symptom for the elderly and women, and deteriorates their quality of life [1,2,3]. Moreover, numerous medicines have side effect of constipation in patients [4,5,6,7]. Folium Sennae (FS), the leaves of *Cassia angustifolia* V_AHL_ (Fabaceae), is a popular laxative (Senna) worldwide in clinical practice and also a common component in the food supplements claimed for weight control [8,9,10,11]. FS contains dianthrone glycosides, namely sennosides A and B, together with other anthraquinones, such as rhein, aloe-emodin and chrysophanol [8,12], which have shown beneficial biological effects including anti-microbial, anti-fungal, anti-inflammatory, anti-oxidant, anti-diabetic and anti-tumor activities [13,14,15,16].

In recent decades, drug transporters have well been recognized as crucial determinants governing the absorption, distribution and excretion of drugs as well as playing important roles in drug-drug, supplement-drug and food-drug interactions [17,18,19,20,21,22,23]. FS and its polyphenolic anthraquinones have exhibited modulations on a number of drug transporters, such as P-glycoprotein (P-gp) [24,25], organic anion-transporting polypeptides (OATPs) and organic anion transporters (OATs), which were reported by in vitro studies [23,26,27]. However, in recent decades there was a consensus that polyphenols were rapidly and extensively metabolized, then mainly existed as conjugated metabolites in the circulation and organs [28,29,30]. Moreover, our pharmacokinetic study of FS reported that the major molecules in the plasma were rhein, rhein sulfate/glucuronide (S/G) and aloe-emodin S/G, which were all acids existing as anions in the serum [24]. The transports of these anionic metabolites were often mediated by modulate multidrug resistance-associated protein 2 (MRP 2) and breast cancer resistance protein (BCRP) [24,31]. Therefore, we hypothesized that these acidic metabolites of FS might modulate MRPs and BCRP. 

MRPs are distributed widely in the body, and transport a number of anticancer drugs such as methotrexate (MTX), vinblastine, 5-fluorouracil, doxorubicine and etoposide [32,33,34]. BCRP is located in brain, breast cells, liver, intestine, kidney, placenta, amd testis [19], and transports a number of anticancer drugs such as MTX, daunorubicin, doxorubicin, gefitinib, imatinib, ireinotecan, mitoxantrone and sunitinib [35]. This study investigated the effect of FS on the pharmacokinetics of MTX, a dicarboxylate immunosuppressant and anticancer drug used as a probe substrate of MRPs/BCRP, in rats [36,37]. Furthermore, cell lines were employed to verify the transporter-based relevant mechanisms.

## 2. Results

### 2.1. FS-MTX Pharmacokinetic Interaction and Relevant Mechanisms

#### 2.1.1. Effect of FS on MTX Pharmacokinetics in Rats

**Figure 1:**
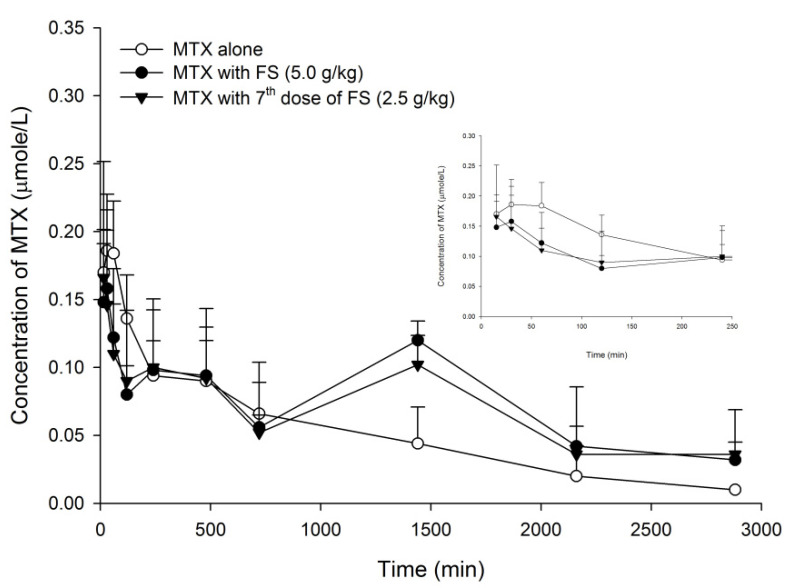
Mean (±S.E.) serum concentration-time profiles of MTX after oral MTX alone (5.0 mg/kg, ○) and with single dose of FS (5 g/kg, ●), and the 7^th^ dose of FS (2.5 g/kg, ▼) (*n* = 5 in each group).

The serum MTX profiles after oral dosing of MTX without and with single dose of 5.0 g/kg of FS and the seventh dose of 2.5 g/kg of FS are shown in Figure 1. The pharmacokinetic parameters of MTX after various treatments are listed in Table 1. When 5.0 g/kg of FS was coadministered, AUC_720–2880_, AUC_0–2880_ and MRT of MTX were significantly increased by 102, 45 and 42%, respectively, whereas AUC_0–240_ was decreased by 26%, and C_max_ was not affected. On other hand, after pretreatment with seven doses of FS (2.5 g/kg), the AUC _720–2880_ and MRT of MTX was significantly increased by 78 and 42%, respectively, whereas AUC_0–240_ was decreased by 25% and C_max_ was not affected.

#### 2.1.2. Characterization of FSM

In order to mimic the real molecules present in the kidney, we have prepared FSM from rats. Characterization of FSM showed that for the study of influence on BCRP activity, the concentrations of rhein, rhein S/G and aloe-emodin S/G were 14.5, 7.2 and 0.6 μM, respectively; on the other hand, for the study of influence on MRP 2 activity, the concentrations of rhein, rhein S/G and aloe-emodin S/G were 25.0, 14.0 and 4.5 μM, respectively.

#### 2.1.3. Effects of FS, FSM and Rhein on BCRP Activity

**Figure 2:**
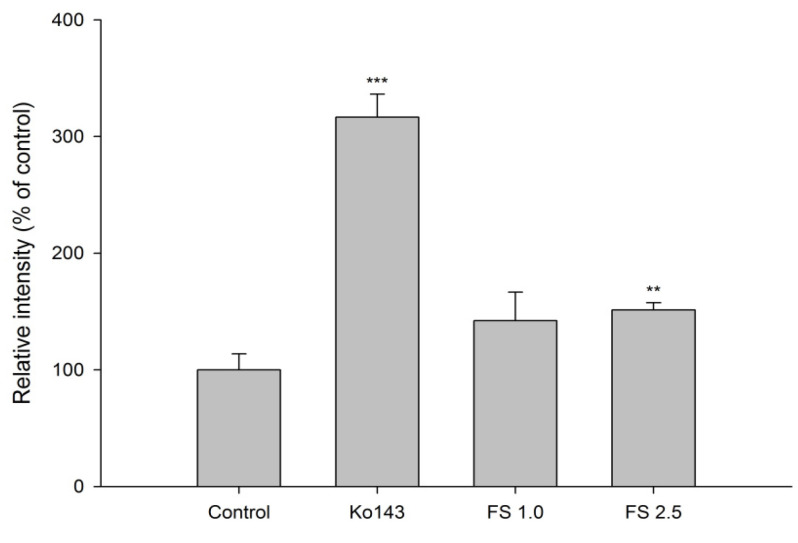
Effects of FS (1.0 and 2.5 mg/mL) and Ko143 (0.5 μM) on the intracellular accumulation of MXR in MDCKII-BCRP cells. ** *p* < 0.01 and *** *p* < 0.001.

**Figure 3:**
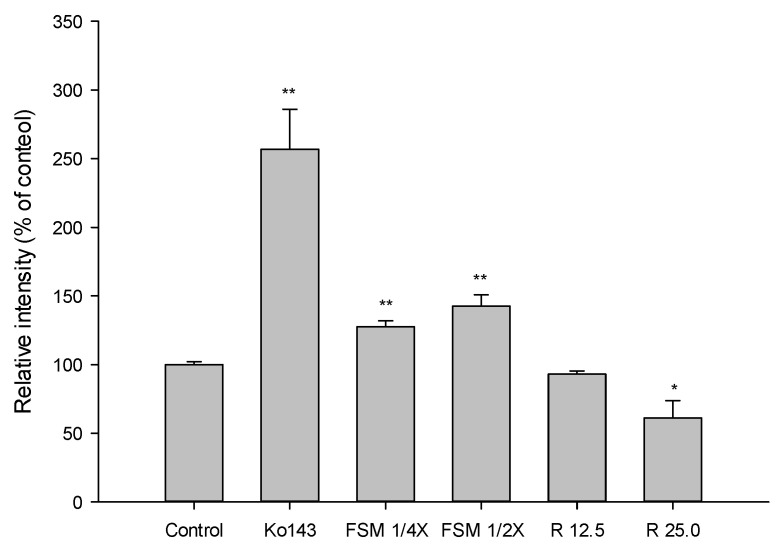
Effects of FSM (1/4 and 1/2-fold serum concentrations), rhein (R, μM) and Ko143 (0.5 μM) on the intracellular accumulation of MXR in MDCKII-BCRP cells. * *p* < 0.05 and ** *p* < 0.01.

The accumulation of MXR in MDCKII-BCRP cells measured after 30-min incubation with tested agents is shown in Figure 2 and Figure 3. Figure 2 shows that FS at 2.5 mg/mL significantly enhanced the intracellular accumulation of MXR by 51%. As a positive control of BCRP inhibitor, Ko143, at 0.5 μM significantly enhanced the intracellular accumulation of MXR by 216%. Figure 3 indicates that when compared to those of blank serum specimen at corresponding concentrations, FSM at 1/4- and 1/2-fold serum concentrations markedly enhanced the intracellular accumulation of MXR by 27 and 42%, respectively. Conversely, 25 μM of rhein reduced the intracellular accumulation of MXR by 33.7%. As a positive control of BCRP inhibitor, Ko143 at 0.5 μM significantly enhanced the intracellular accumulation of MXR by 157%.

#### 2.1.4. Effect of FSM and Rhein on MRP 2 Activity

**Figure 4:**
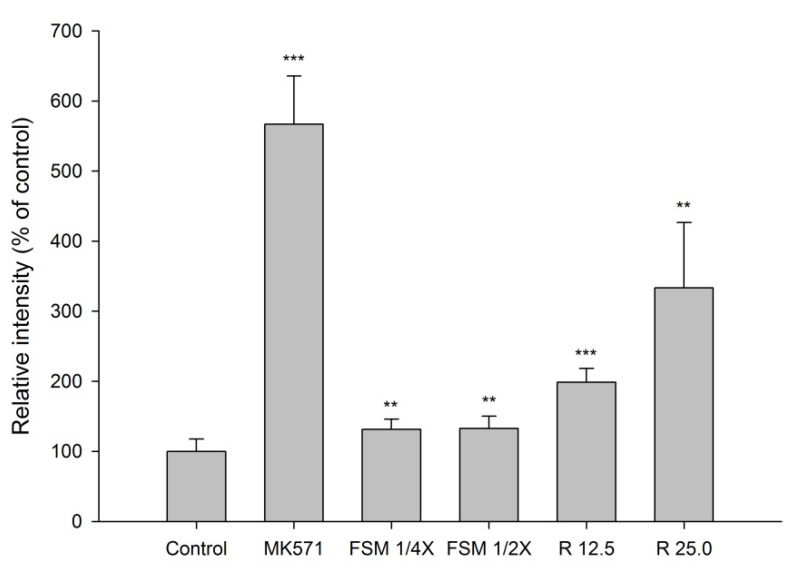
Effects of FSM at 1/4 and 1/2-fold serum concentrations, rhein (R, μM) and MK571 (10 μM) on the intracellular accumulation of GSMF in MDCK II cells. ** *p* < 0.01 and *** *p* < 0.001.

The accumulation of GSMF in MDCKII cells measured after 30-min incubation with tested agents is shown in Figure 4. The results indicate that FSM at 1/4- and 1/2- fold serum concentrations significantly increased the intracellular accumulation of GSMF by 31% and 33 %, respectively. In addition, 12.5 and 25 μM of rhein increased the intracellular accumulation of GSMF by 99 and 233%, respectively. As a positive control of MRP 2 inhibitor, MK571 at 10 μM significantly increased the intracellular accumulation of GSMF by 467%.

## 3. Discussions

In the present study, MTX was selected as a probe for evaluating the influence of FS on the pharmacokinetics of MRPs/BCRP substrates. A seven-dose regimen of FS was intentionally designed to mimic the long-term use of FS. The results show that a single dose of FS (5.0 g/kg) increased the AUC_0–2880_, AUC_720–2880_ and MRT of MTX, and the seventh dose of FS (2.5 g/kg) enhanced the AUC_720–2880_ and MRT of MTX, indicating that both regimens of FS decreased the elimination of MTX. In addition, the AUC_720–2880_ were significantly increased by 102 and 78% following the coadministrations with single dose at 5.0 g/kg and repeated dose at 2.5 g/kg, respectively, indicating that FS decreased the elimination of MTX in a dose-dependent manner.

As shown in Figure 1, the profiles of MTX following co-administrations of FS showed prominent second peaks at 1440 min, which caused the elimination constants and half lives of MTX to be incalculable. That was why the parameters of elimination constants and half lives of MTX were not provided in Table 1. On other hand, the amounts of MTX excreted in urine were not measured in this study, and the inhibition on MTX excretion via the kidney could not be evaluated.

Based on the known pharmacokinetic of FS, the polyphenolic anthranoids were rapidly and extensively metabolized to conjugated metabolites [24]. For mimicking the virtual molecules interacting with MRP 2 and BCRP in the kidney, we had prepared FSM from rats, and the characterization of FSM showed that it contained rhein, rhein S/G and aloe-emodin S/G, mainly rhein, which were all acids existing as anions in the bloodstream and probable inhibitors of MRP 2/BCRP [38,39]. The results of transport assay showing that FSM increased the intracellular accumulation of GSMF (a substrate of MRP 2) and MXR (a substrate of BCRP) indicated that FSM inhibited MRP 2 and BCRP. Rhein, a major metabolite of sennoside A and B, was also evaluated by transport assay. Rhein showed inhibition on MRP 2 like FSM. However, regarding the modulations on BCRP, rhein showed activation, which was opposite to the inhibition by FSM. We assumed that the conjugated metabolites of polyphenols in FSM might overwhelm the activation effect of rhein, and resulted in a net effect of inhibition on BCRP. Taken together, these ex-vivo findings indicated that FSM inhibited MRP 2- and BCRP-mediated excretions of MTX in the kidney, which could in part explain the increased systemic exposure of MTX.

Concerning the absorption of MTX, observing the profiles closely found that the absorptions of MTX (shown in the right upper diagram in Figure 1) were similarly decreased by single-dose and repeated-dose regimens of FS. The early exposures (AUC_0–240_) of MTX were slightly decreased, but did not reach statistical significance. The results of the transport assay showed that FS inhibited BCRP. However, as a major constituent in FS [24], rhein showed activation on BCRP, which was opposite to FS. We speculated that it is because rhein was also a metabolite of sennosides A and B via micofloral biotransformation, and the concentration in the intestine was increased to become greater than FS [24,40,41]. Therefore, rhein might override FS and result in a net effect of activation on BCRP, which would lead to decreased absorption of MTX. In this study, although the absorption of MTX was not significantly decreased by FS, it clearly demonstrated that rhein was an activator of BCRP.

Taken together, during the absorption phase, rhein activated BCRP to decrease the absorption of MTX, which was offset by the inhibition of BCRP due to FS, resulting in non-significant decrease in MTX absorption. On other hand, during the elimination phase, FSM decreased the elimination of MTX through inhibitions on MRP2 and BCRP, which overwhelmed the activation of BCRP by rhein. To sum up, that FSM inhibited the MRP 2- and BCRP- mediated excretion of MTX could explain the increased bioavailability of MTX in rats.

A number of clinically important anticancer drugs are verified as substrates of MRP 2 and/or BCRP, such as vinblastine, 5-fluorouracil, doxorubicine, etoposide, daunorubicin, gefitinib, imatinib, ireinotecan, mitoxantrone and sunitinib [32,33,35]. The pharmacokinetics of these MRP 2/BCRP substrates are very likely altered by concurrent use of FS. We suggest that when patients are treated with these anticancer agents, if FS is used at the same time, the adverse effects of anti-cancer drugs should be carefully monitored to ensure the safety of chemotherapy.

## 4. Materials and Methods

### 4.1. Chemicals and Reagents

The crude drug of FS was purchased from an herbal drugstore in Taichung, Taiwan and identified by Dr. Yu-Chi Hou. MTX (25 mg/mL) was obtained from Pharma B. V. (Haarlem, The Netherlands). TDxFLx methotrexate monoclonal whole blood reagent pack was obtained from Abbott Laboratories (Abbott Park, IL, USA). Sennoside A (purity 96%), aloe-emodin (purity 95%), dimethyl sulfoxide (DMSO), sodium dodecyl sulfate (SDS), Triton X-100, verapamil (purity 99 %), β-glucuronidase (Type B-1, from bovine liver) and sulfatase (type H-1 from *Helix pomatia*, containing 14,000 units/g of sulfatase and 498,800 units/g of β-glucuronidase) were purchased from Sigma Chemical Co. (St. Louis, MO, USA). Rhein (purity 95%) was purchased from Aldrich Chemical Co. (Milwaukee, WI, USA). 1,8-Dihydroxyanthraquinone (purity 98%) and amyl paraben (purity 98%) were obtained from Tokyo Chemical Industry Co. (Tokyo, Japan). Methanol, acetonitrile, glacial acetic acid (99%) and ethyl acetate were LC grade and purchased from J.T. Baker, Inc. (Phillipsburg, NJ, USA). L(+)-Ascorbic acid (purity 99.7%) was obtained from RdH Laborchemikalien GmbH & Co. KG (Seelze, Germany). Other reagents were HPLC grade or analytical grade. 3-(4,5-Dimethylthiazol-2-yl)-2,5-diphenyltetrazolium bromide (MTT) (purity 98%) was obtained from Alfa Aesar (Lancaster, UK). Fetal Bovine Serum (FBS) was supplied by Biological Industries Inc. (Kibbutz, Beit Haemek, Israel). Penicillin-Streptomycin-Glutamine (PSG), 5-chloromethylfluorescein diacetate (CMFDA), Dulbecco’s modified Eagle medium (DMEM), trypsin/EDTA, Hank’s buffered salt solution (HBSS) and 4-(2-hydroxyethyl)-1-piperazineethanesulfonic acid (HEPES) were purchased from Invitrogen (Carlsbad, CA, USA). MK 571, mitoxantrone (MXR, purity 97%) and Ko143 (purity 96%) were obtained from Enzo Life Sciences, Inc. (Farmingdale, NY, USA). Milli-Q plus water (Millipore, Bedford, MA, USA) was used throughout this study.

### 4.2. Preparation and Characterization of FS Decoction

The preparation and quantitation of FS decoction had been published in our previous study and briefly described as follows [24]. Three hundred microliters of FS decoction (1.0 g/mL) was mixed with 700 μL of methanol. After centrifugation, the supernatant (180 μL) was mixed with 20 µL of amyl paraben methanol solution (50.0 µg/mL, as internal standard) and 20 µL was subject to HPLC analysis. The mobile phase consisted of methanol (A) – 0.1 % acetic acid (B) and eluted in a gradient manner as the timetable (min, %B):(0, 55), (5, 55), (8, 48), (12, 35), (22, 20), (30, 20), (32, 55) (35, 55). The detection wavelength was set at 270 nm and the flow rate was 1.0 mL/min.

### 4.3. Animals and Drug Administration

All animal experiments were carried out in strict accordance with the recommendations by “The Guidebook for the Care and Use of Laboratory Animals” published by the Chinese Society for the Laboratory Animal Science, Taiwan. The experimental protocol had been reviewed and approved by the Insititutional Animal Care and Use Committee of China Medical University. Male Sprague-Dawley rats weighing 320–465 g were purchased from BioLASCO Taiwan Co., Ltd. (Yilan, Taiwan) and housed in a conditioned environment with 12-h light/dark cycles. Food and water were supplied ad libitum until 12 h prior to experiment. The rats fasted overnight before drug administration and food was offered 3 h after FS dosing. MTX was diluted with water to afford a concentration of 2.5 mg/mL. Three groups of rats were orally given MTX (5.0 mg/kg) alone, and with a single dose of FS (equivalent to 5.0 g/kg of FS) and seven consecutive doses of FS (equivalent to 2.5 g/kg of FS), respectively, in a parallel design. FS decoction was administered 30 min before MTX dosing via gastric gavage in the treatment groups.

### 4.4. Blood Collection and Determination of Serum MTX Concentration

Blood samples were collected at 15, 30, 60, 120, 240, 480, 720, 1440, 2160, 2880 min after MTX dosing. At each time point, 0.5 mL of blood was withdrawn. The blood samples were collected in microtubes and centrifuged at 10,000× *g* for 10 min to obtain serum, which was stored at −20 °C until analysis. The concentrations of MTX in serum were determined using a specific monoclonal fluorescence polarization immunoassay (FPIA). Validation of the calibration curve was carried out by testing three controls with low, medium and high concentrations before sample analysis. The assay was calibrated for concentrations from 0 to 1.0 μM and the lower limit of quantitation was 0.02 μM.

### 4.5. Cell Line and Culture Conditions

Madin-Darby canine kidney type II (MDCKII) cells expressing BCRP (MDCKII-BCRP) and MDCKII-wild type (WT) cells were kindly provided by Prof. Dr. Piet Borst (Netherlands Cancer Institute, Amsterdam, The Netherlands).

MDCKII-WT and MDCKII-BCRP cells were grown in DMEM medium supplemented with 10% FBS, 100 units/mL of penicillin, 100 μg/mL of streptomycin and 292 μg/mL of glutamine at 37 °C in a humidified incubator containing 5% CO_2_.

The medium was changed every other day and cells were subcultured when 80% to 90% confluency was reached.

### 4.6. Cell Viability Assay

The effects of tested drugs on the viability of cells described above were evaluated by MTT assay [42]. Cells were seeded into a 96-well plate. After overnight incubation, the tested agents were added into the wells and incubated for 24 h, then 15 μL of MTT (5.0 mg/mL) was added into each well and incubated for 4 h. In this period, MTT became formazan crystal by live cells. Following removal of the supernatant, SDS solution (20%) was added to dissolve the purple crystal at the end of incubation and the optical density was detected at 570 nm by a microplate reader (BioTex, Highland Park, Winooski, VT, USA).

### 4.7. Preparation and Characterization of Serum Metabolites of FS (FSM)

In order to mimic the molecules interacting with MRP 2 and BCRP in kidney cells in vivo, FSM was prepared from rats and characterized. Briefly, FS decoction was orally administered to rats fasted overnight. Blood was collected at 10 min after dosing. After coagulation, the serum was vortexed with 3-fold methanol. After centrifugation, the supernatant was concentrated in a rotatory evaporator under vacuum to dryness. To the residue, an appropriate volume of water was added to afford FSM solution with 10-fold serum concentration, which was divided into aliquots and stored at −80 °C for later use.

In order to characterize FSM, 100 μL of FSM solution was mixed with 50 μL of sulfatase (1000 units/mL in pH 5 acetate buffer), 50 μL of ascorbic acid (100 mg/mL), and incubated at 37 °C for 30 min. After hydrolysis, the serum was acidified with 50 μL of 0.1 N HCl and partitioned with 250 μL of ethyl acetate (containing 0.2 μg/mL of 1, 8-dihydroxyanthraquinone as internal standard). The ethyl acetate layer was evaporated under N_2_ to dryness and reconstituted with an appropriate volume of methanol prior to HPLC analysis, which was following a method reported previously [24]. On the other hand, blank serum was also collected from rats and processed in the same manner as FSM to prepare blank specimens as controls for comparison with correspondent specimens of FSM.

### 4.8. Effects of FS, FSM and Rhein on BCRP Activity

The transport assay was performed to evaluate the effects of FS and FSM on BCRP activity. MDCKII-BCRP cells were suspended at a density of 1 × 10^6^ in each reaction tube. MXR was used as a probe for evaluating the effects of FS, FSM and rhein on BCRP-mediated transport. Ko143 was used as a positive control of the BCRP inhibitor. Before transport assay, MDCKII-BCRP cells were pre-incubated with tested agents (FS, FSM, rhein and Ko143) at 37 °C for 15 min. MXR was then added and co-incubated for another 30 min. Finally, ice-cold PBS was used to wash the cells. After centrifugation, the supernatant was removed and the cell pellet was re-suspended by ice-cold PBS. Subsequently, the fluorescence of MXR was determined by a FACScan flow cytometer (Becton Dickinson Immunocytometry Systems, San Jose, CA, USA) equipped with a standard HeNe laser. All transport studies were performed in triplicates.

### 4.9. Effect of FSM and Rhein on MRP 2 Activity

The transport assay was conducted to measure the effects of FSM and rhein on MRP 2 activity as previously described [43,44,45]. Briefly, MDCKII cells (1 × 10^5^ cells/well) were cultured in each well of a 96-well plate. After overnight incubation, the medium was removed and washed three times with ice-cold PBS buffer. The tested agents (CMFDA plus FSM or CMFDA plus rhein) were added into each well and incubated at 37 °C. After 30-min incubation, the supernatants were removed and washed for three times with ice-cold PBS. Subsequently, 100 μL of 0.1 % Triton X-100 was added to lyse the cells, and the fluorescence was measured with excitation at 485 nm and emission at 528 nm.

To quantitate the content of protein in each well, 10 μL of cell lysate was added to 200 μL of diluted protein assay reagent (Bio-Rad, Hercules, CA, USA) and the optical density was measured at 570 nm. The relative intracellular accumulation of glutathione-methylfluorescein (GSMF, a metabolite of CMFDA), a fluorescent substrate of MRP 2, was calculated by comparing it with that of control after protein correction.

### 4.10. Data Analysis

The serum concentration of MTX was analyzed using noncompartment model of WinNonlin (version 6.4, Pharsight Co., Cary, NC, USA). The peak serum concentration (C_max_) was obtained from experimental observation. The area under the serum concentration-time curve (AUC_0__–t_) was calculated using trapezoidal rule to the last point. Unpaired Student’s t-test was used to analyze the difference between two groups and one-way ANOVA with Scheffe test was used for statistical comparison among three groups taking *p* < 0.05 as significant.

## 5. Conclusions

Concurrent use of FS increased the systemic exposure and MRT of MTX through inhibitions on MRP 2 and BCRP.

## Figures and Tables

**Table 1:** Pharmacokinetic parameters of MTX after oral MTX alone (5.0 mg/kg) and with single dose of 5.0 g/kg of FS, and the 7^th^ dose of 2.5 g/kg of FS.

	Parameters	MTX Alone		MTX + FS (5.0 g/kg, Single Dose)		MTX + FS (2.5 g/kg, 7 Doses)	
Treatments							
C_max_		0.2	±0.0_2_	0.2	±0.0_2_	0.2	±0.0_3_
AUC _0–240_		32.9	±3.0	24.3	±2.5	24.8	±4.5
				(−26%)		(−25%)	
AUC _720–2880_		73.4	±14.9 ^a^	148.3	±15.6 ^b^	131.7	±4.8 ^b^
				(+102%)		(+78%)	
AUC _0–2880_		147.1	±15.6 ^a^	213.7	±18.6 ^b^	196.2	±10.4 ^ab^
				(+45%)		(+33%)	
MRT _0–2880_		841.9	±103.6 ^a^	1199.2	±68.3 ^b^	1195.8	±88.6 ^b^
				(+42%)		(+42%)	

Data expressed as mean ± S.D. ^a, b^ Significant difference between means are denoted by different letters (*p* < 0.05). C_max_ (μmol/L): the peak serum concentration. AUC _0–240_ (μmol·min/L): area under concentration-time curve from 0 to 240 min. AUC _720–2880_ (μmol·min/L): area under concentration-time curve from 720 to 2880 min. AUC _0–2880_ (μmol·min/L): area under concentration-time curve to 2880 min. MRT (min): mean residence time.

## Data Availability

The raw data presented in this study will be shared on request from the corresponding author.
